# Coronally Advanced Flap with Connective Tissue Graft for Treating Orthodontic-Associated Miller Class III Gingival Recession of the Lower Incisors: A One-Year Retrospective Study

**DOI:** 10.3390/jcm11010235

**Published:** 2022-01-01

**Authors:** Evgeny Weinberg, Roni Kolerman, Lazar Kats, Omer Cohen, Daya Masri, Alon Sebaoun, Gil Slutzkey

**Affiliations:** 1 Departments of Periodontology and Oral Implantology, Departments of Oral Biology, Goldschleger School of Dental Medicine, Sackler Faculty of Medicine, Tel Aviv University, Tel Aviv 6997801, Israel; 2 Department of Periodontology and Oral Implantology, Goldschleger School of Dental Medicine, Sackler Faculty of Medicine, Tel Aviv University, Tel Aviv 6997801, Israel; kolerman@netvision.net.il (R.K.); omerco2@gmail.com (O.C.); alon.sebaoun@gmail.com (A.S.); slutzkey@gmail.com (G.S.); 3 Department of Oral Pathology, Oral Medicine and Maxillofacial Imaging, Goldschleger School of Dental Medicine, Sackler Faculty of Medicine, Tel Aviv University, Tel Aviv 6997801, Israel; lazarkat@tauex.tau.ac.il; 4 Rabin Medical Center, Department of Oral and Maxillofacial Surgery, Petach-Tikva 4941492, Israel; dr.dayamasri@gmail.com

**Keywords:** gingival recession, Miller class III, mandibular incisors, post-orthodontic

## Abstract

(1) Background: To assess the clinical outcome of coronally advanced flap combined with connective tissue graft for the treatment of orthodontic-associated Miller Class III gingival recession of the lower incisors. (2) Methods: This study included 15 patients who had undergone orthodontic treatment prior to development of recession. Measurements of recession depth, recession width, probing depth, and width of keratinized tissue were performed clinically immediately before surgery and after one year. In addition, digital measurements of recession depth, recession width, and root coverage esthetic score were performed on intraoral photographs. (3) Results: Significant reduction was observed for probing depth, recession depth, and recession width at one year, with significant increase in width of keratinized tissue. Mean root coverage was 83 ± 24% for recession depth, while complete root coverage was achieved in 10 out of 21 recessions (48%). The average root coverage esthetic score at 12 months was 7.1 ± 2.6. An interaction was found between initial recession depth and mean root coverage. (4) Conclusions: Within the limitations of this study, our results confirm that combination of coronally advanced flap and connective tissue graft is effective in reducing post-orthodontic Miller Class III recessions of the mandibular incisors, even when the correction of the tooth malposition, is unattainable.

## 1. Introduction

Gingival recession (GR) is the displacement of the soft tissue margin apical to the cemento-enamel junction (CEJ), which leads to root surface exposure to the oral environment [1]. Untreated buccal GR is associated with thermal and tactile sensitivity, esthetic complaints, and potential complications in maintaining optimal oral hygiene [2]. Moreover, untreated GR has a tendency for further apical displacement over time despite high patient self-care motivation, particularly when the keratinized tissue is reduced or absent [3].

A growing body of evidence relates orthodontic treatment to the development of GR [2,4,5,6]. Experimental studies suggest that movement of the root beyond the envelope of the alveolar process causes stretching and thinning of the gingiva, which increases the risk of GR [7,8], particularly in patients with reduced gingival thickness [5,9]. Mandibular incisors seem to be the most vulnerable teeth to the development of post-orthodontic GR [5,10,11] due to a tooth–arch relationship that results in labially prominent teeth covered with a thin or nonexistent labial plate of bone and relatively thin marginal gingiva [12]. In addition, mandibular lingual fixed retention may be associated with a higher prevalence of GR, especially in proclined lower incisors [10,13]. Another possible cause of post-orthodontic GR in mandibular incisors may be tooth malposition due to an unwanted force extracted by distortion and activation of the retainer’s wire [14].

If malposition of teeth with roots positioned outside the alveolar envelope is the supposed etiology for GR, then orthodontic treatment should be considered with or without periodontal therapy [15]. However, orthodontic retreatment can be performed only when there is enough lingual bone support, assessed by computerized tomography (CBCT) [16]. Moreover, patients often refuse orthodontic correction of roots positioned outside the alveolar envelope, since this type of orthodontic movement requires reinsertion of a fixed orthodontic appliance in the lower arch for several months. In these cases, surgical root coverage can be considered despite the tooth malposition.

Although numerous surgical approaches for root coverage have been reported, the coronally advanced flap (CAF) together with connective tissue graft (CTG) is still considered the gold standard in Miller Class I or II buccal recessions in terms of clinical outcomes [17,18,19]. However, limited data is available regarding the Miller Class III recessions [20], particularly in orthodontic-associated GRs of the lower incisors. In such cases, the interdental periodontal support loss is mild to moderate, soft tissue loss in the interdental area is present, or a malpositioning of the teeth prevents the attempt of full root coverage [21].

To help address this lacuna, the objective of this retrospective study was to assess the clinical outcome of CAF combined with CTG for the treatment of orthodontic-associated Miller Class III gingival recession of the lower incisors, one-year post-treatment.

## 2. Materials and Methods

The study design was approved by the Institutional Ethics Committee of Tel Aviv University (IEC No. 0002616-1). Data were collected by retrospective evaluation of all consecutive patients that met the inclusion criteria.

The patients were treated by one experienced periodontist (EW) between January 2018 and December 2019. All patients signed an informed consent concerning the planned treatment and received detailed information about alternative options.

The study included 15 patients (2 males, 13 females), aged 19–29 years (23.87 ± 3.2), in whom 21 sites were treated. The mean period between completion of orthodontic treatment and the surgical root coverage was seven years (ranging from 1 to 11). 

Inclusion criteria were patient age ≥18 years, non-smoking status, and absence of any systemic disease that may affect periodontal treatment.

Exclusion criteria consisted of medically compromised patients, pregnant women, smokers, history of periodontal disease, previous mucogingival procedures including frenectomy, and/or poor hygiene levels (full-mouth plaque score more than 20%). Patients with non-identifiable cemento-enamel junction and those who underwent orthodontic correction of malposed incisors prior to surgery were excluded as well.

All patients presented a buccal Miller Class III gingival recession (Cairo recession Type 2 (RT2)) of the lower incisors, had undergone orthodontic treatment prior to development of recession, and had complete clinical records and intraoral photographs. In addition, all patients presented with bonded lingual retainer which was not replaced all over the study and tooth malposition being either buccal inclinations or the position of the roots outside the alveolar envelope. Minimal recession depth was 2 mm.

### 2.1. Initial Therapy

Prior to the surgery, all patients underwent oral hygiene instruction, including atraumatic toothbrushing using modified Bass technique, as well as full-mouth supra- and subgingival scaling and professional tooth cleaning with the use of a rubber cup and low abrasive polishing paste. Surgical treatment of the recession defects was not scheduled until the patient demonstrated an adequate standard of supragingival plaque control.

### 2.2. Surgical Procedures

**Figure 1:**
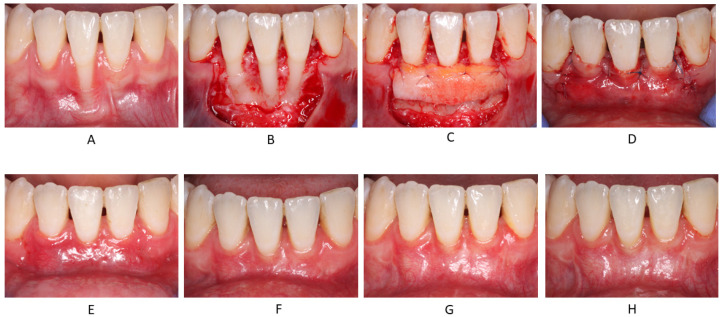
Representative case: (**A**) Pre-op; (**B**) Flap elevation; (**C**) Fixation of CTG at CEJ level; (**D**) Closure of CAF above the CEJ; (**E**) 2 weeks follow-up; (**F**) 1 month follow-up; (**G**) 3 months follow-up; (**H**) 12 months follow-up.

The surgical technique for gingival recession coverage was the trapezoidal-type of CAF [22], fully covering a CTG obtained by means of de-epithelialization of a free gingival graft (FGG) [23] (Figure 1). 

The recipient site was prepared using a #15C blade by a variable (split–full–split) thickness trapezoidal flap extending to the alveolar mucosa that was elevated, and the roots were exposed. To give the CAF a vertical dimension and close adaptation of the alveolar bone and root surfaces, the labial submucosal tissue (LST) was isolated and removed by means of two incisions: one deep, to detach LST from the periosteum, and one superficial, to separate the LST from the inner surface of the alveolar mucosa [24]. FGG was harvested from the canine to first molar area of the palate, de-epithelialized using a #15C blade extraorally, and trimmed to a thickness of approximately 2 mm. The size of the graft was adjusted to cover all the involved teeth. After mechanical treatment of the exposed root surface with Mini-Five™ Gracey curette (Hu-Friedy, Chicago, IL, USA), the CTG was positioned at the level of the CEJ and sutured with single stiches using 7-0 resorbable suture (Vicryl^®^, Ethicon; Johnson & Johnson, Somerville, NJ, USA) to the de-epithelized papilla. The flap was then positioned at least 1 mm coronally to the CEJ [25] and closed using the same 7-0 resorbable suture for single stiches on the lateral release incision and a nonresorbable 6-0 suture material (Prolene, Ethicon) for a coronal sling suture around the teeth.

All patients were administered with antibiotic therapy (875 mg of amoxicillin/clavulanate potassium twice daily or 150 mg of clindamycin four times a day for penicillin-sensitive patients) for one week. Anti-inflammatory analgesics (ibuprofen) were prescribed for five days. Patients were also instructed to avoid brushing the teeth in the treated area but to rinse with chlorhexidine solution (0.2%) twice daily for one minute. Sutures were removed after 14 days. Patients were recalled for control visits once a week in the first month, then monthly up to one-year post-surgery.

Postoperative healing was uneventful in all treated cases and at all follow-up visits (Figure 1). Furthermore, patients reported no events of acute post-operative pain.

### 2.3. Outcome Measurements

The following variables were measured: recession depth (RD), recession width at the CEJ (RW), probing depth (PD), and width of keratinized tissue (KTW). The clinical measurements were performed by the operating periodontist (EW), at the mid-buccal aspect of the root, immediately before surgery (baseline) and at the one-year follow-up visit, with a 1 mm calibrated periodontal probe (PCP-UNC 15, Hu-Friedy). In addition, digital measurements of RD and RW were performed by an independent evaluator (GS), using ImageJ software (https://imagej.net/, ImageJ, RRID:SCR_003070, accessed on 12 March 2021) on magnified intraoral images (X10) photographed at the baseline and at the one-year follow-up visit using a DSLR camera. To facilitate the subsequent analysis, the photographs were centered to the contact region of the lower central incisors at the midline. All the pictures were taken by the same operator (EW). Only photographs with near ideal ortho-radial positioning were included in the study [26]. The digital measurements were calibrated to the true value using the same periodontal probe photographed aligned parallel to the buccal surface of the right central mandibular incisor. Digital measurements were repeated after a minimum of 14 days for all patients and recessions, enabling calculation of the method’s error.

The esthetic outcome was evaluated using the root coverage esthetic score (RES) system [27] on intraoral photographs, with the same magnification as for the clinical measurements, by one operator (GS). The maximum esthetic score was 10.

### 2.4. Statistical Analysis 

The inter-examiner reproducibility of the clinical and digital measurements of RD and RW, as well as intra-examiner reproducibility of all the digital measurements, was assessed by interclass correlation coefficient. Both descriptive statistics and analysis of variance (ANOVA) were performed to compare the baseline and 12-month outcomes. ANOVA with repeated measures was used to find interactions between the changes from baseline to 12 months in RD, RW, KTW (ΔRD, ΔRW, ΔKTW, respectively), and percentage of root coverage (%RC), and the different baseline parameters. A correction by the Benjamini–Hochberg procedure was applied for multiple correlations between the different parameters. The analysis was performed using SPSS version 25.0 (IBM, Armonk, NY, USA).

## 3. Results

Significant reduction was observed for PD, RD, and RW at one-year post-treatment, compared to the baseline measurements, with significant increase in KTW (Table 1).

Mean root coverage was 83 ± 24% for the recession depth, while complete root coverage was achieved in 10 out of 21 recessions (48%) (Table 2).

The average root coverage esthetic score at 12 months was 7.1 ± 2.6 (Table 3).

An interaction was found for ΔRD (mm; dependent variable) to RD and KW at baseline (independent variables). However, after linear regression and Benjamini–Hochberg correction for multiple comparisons, only RD at baseline had an interaction with ΔRD. When %RC, ΔRW, and ΔKTW were the dependent variables, no interactions were found.

The inter- and intra-examiner measure of agreement was Cohen’s Kappa = 1, for both clinical and photographical measurements, which indicates a high level of internal consistency.

## 4. Discussion

The purpose of the present retrospective study was to evaluate the clinical and esthetic outcomes of the CAF with CTG in the treatment of post-orthodontic Miller Class III gingival recession of the lower incisors. The study included 15 patients who had undergone orthodontic treatment prior to development of recession and for whom orthodontic correction was not feasible, since the incisors were in malposition due to patient objection to corrective procedures or given the absence of lingual bone support as revealed by CBCT.

Most of the patients had completed the orthodontic treatment as young adults (between the ages of 14 and 18 years); however, accurate data could not be obtained regarding the exact starting time of the recession and the changes over time. All the patients reported no recession at the completion of orthodontic treatment. Recent fast progression of the recession caused them great concern and encouraged them to seek treatment urgently.

In this study, we classified gingival recessions using the Miller classification [21], probably the most commonly employed system for this purpose. Cairo and colleagues had suggested another classification system of gingival recessions, based on the interproximal clinical attachment level [28]. According to this classification, all the treated Miller Class III recessions in this study were RT2. However, in Cairo and colleagues’ classification, the malposition of the root is not considered a prognostic criterion, although it is a limiting factor for the amount of root coverage achieved at the buccal site after surgery [14]. This may be associated with the blood supply provided by interproximal soft tissue to the buccal flap/graft during the healing process. Therefore, in cases of post-orthodontic lower incisors recessions, the Miller classification, which takes into consideration the tooth position, is more appropriate.

Applying CAF combined with the CTG technique in Miller Class I or II buccal recession-type defects affecting the lower incisors, Zucchelli and colleagues reported up to 98% mean root coverage with 88% complete root coverage (CRC), provided the labial submucosal tissue was removed from the inner surface of the alveolar mucosa [24]. However, the scholarship on Miller Class III recessions is quite limited, mostly focused on the comparison of techniques or the evaluation of results obtained by new procedures [29,30,31].

In the present evaluation, mean root coverage for Miller Class III orthodontic-associated gingival recessions was found to be 83 ± 24%, while CRC was recorded in 48% of the sites at one year compared to baseline measurements. RD, RW, and PD were significantly reduced; however, only RD at baseline had demonstrated interaction with ΔRD. Interestingly, no interaction was found for %RC, RW, and KTW, pointing to the RD as the main prognostic factor for the root coverage.

A case series study evaluating CAF with CTG for the treatment of Miller Class III gingival recessions in mandibular central incisors found 86% of mean root coverage with 43% of CRC [29]. Interestingly, an additional study by the same authors using CTG in combination with tunnel technique showed only 74% of mean root coverage and 14% of CRC for Miller Class III defects in mandibular incisors [30]. The investigators explained these differences by facts that minimal efforts were made in coronally advancing the flap with the tunnel technique and that most of the root coverage occurred through vascularization of the exposed graft.

A recent study comparing clinical outcomes of CTG with and without enamel matrix derivative in the treatment of Miller Class III defects on mandibular anterior teeth reported 78 and 73% mean root coverage and 22 and 18% of CRC, respectively, 12-months post-treatment [31]. Another recent study presented results of modified tunnel double papilla procedure for root coverage of post-orthodontic Miller Class III recessions with either the removal or not of the bonded lingual retainer (BLR) [14]. An improvement of 43% was found without the removal of the BLR, compared to 87% once the BLR was removed prior to surgery; however, only a small number of cases was evaluated in each group.

Regarding the mean root coverage (83 ± 24%), our results are in line with the findings from the aforementioned studies; however, CRC was slightly higher (48%) in our evaluation. Although CRC was reached in nearly half of the sites only and consequently given a final mean root coverage esthetic score of 7.1 ± 2.6, this parameter may not be considered the most important treatment outcome; this is particularly true regarding non-esthetic areas such as anterior mandible, which is known to have worse results than other regions in the oral cavity [28]. Evidently, a maintainable situation was provided in all cases by creating an adequate zone of keratinized tissue (KTW12 months = 2.6 ± 0.8 mm), thus preventing frenum and muscle pull and allowing easier brushing in this area.

The mean esthetic score in the present study was 7.1 ± 2.6. In a recent meta-analysis, Cairo and colleagues compared esthetic results in different root coverage procedures [32]. The mean esthetic score in the different studies included in the meta-analysis for procedures with a CTG ranged between 7.5 and 9.45. Our results are somewhat lower than their results. However, we evaluated only Miller Class III recessions; since the RES is largely sensitive to the amount of root coverage [27] and Miller Class III recessions are considered highly challenging for CRC [20], the present esthetic score result is satisfactory.

An interesting finding of the present study is the statistically significant PD reduction (2.3 ± 1.3 to 1.0 ± 0.5), aligning with a previous report [24]. This favorable outcome may be due to the baseline PD measurements at the buccal aspect of lower incisors that are greater compared to the mean buccal PD reported in the literature for gingival recession [33]. A possible explanation involves the presence of deep bone dehiscence and a shallow vestibulum depth. In this clinical scenario, a buccal PD apical to the gingival recession forms more frequently, once a deep gingival defect reaches the vestibular fornix. The clinically significant PD and RD reductions, alongside the increase in KTW, represent a meaningful improvement in the periodontal conditions, which could be beneficial for the prognosis of the affected tooth and could justify the treatment of deep gingival recession affecting lower incisors, even in the absence of the traditional esthetic or dentine hypersensitivity indications.

The major limitations of this study are its retrospective nature of analysis, limited sample, absence of information about the long-term stability, and lack of blinded examination of some of the clinical parameters. However, photographic examination by an independent evaluator and a high level of internal consistency partially compensates for some of these drawbacks.

Within the limitations of this study, our results confirm that combination of CAF and CTG may significantly improve post-orthodontic Miller Class III recessions of the mandibular incisors, even when correction of the tooth malposition is unattainable.

## 5. Conclusions

Our findings suggest that combination of coronally advanced flap with connective tissue graft may significantly improve post-orthodontic Miller Class III recessions of the mandibular incisors.

## Figures and Tables

**Table 1:** Mean and SD at baseline and at 12 months of all evaluated parameters.

	Baseline Mean ± SD	12 Months Mean ± SD	*p* Value Baseline vs. 12 Months	Mean Change from Baseline to 12 Months
PD (mm)	2.3 ± 1.3	1.0 ± 0.5	0.0001	−1.3
RD (mm)	4.2 ± 1.7	0.7 ± 0.8	0.0001	−3.5
RW (mm)	3.8 ± 0.8	1.7 ± 1.7	0.0001	−2.1
KTW (mm)	0.7 ± 0.9	2.6 ± 0.8	0.0001	1.9

*p* < 0.05 indicates statistically significant differences. PD, probing depth; RD, recession depth; RW, recession width; KTW, keratinized tissue width.

**Table 2:** Percentage of root coverage and complete root coverage at 12 months.

	12 Months Mean ± SD
Percentage of root coverage (%RC)	83 ± 24%
Complete root coverage (CRC)	10/21 (48%)

**Table 3:** Root coverage esthetic score (RES) at 12 months.

	Mean ± SD
GM	4.4 ± 1.8 (out of 6)
MTC	1.0 ± 0.2 (out of 1)
STT	0.8 ± 0.4 (out of 1)
MGJ	0.7 ± 0.5 (out of 1)
Gingival color	0.4 ± 0.5 (out of 1)
RES	7.1 ± 2.6 (out of 10)

GM, gingival margin level; MTC, marginal tissue contour; STT, soft tissue texture; MGJ, mucogingival junction.

## Data Availability

The datasets analyzed during the current study are available from the corresponding author on reasonable request.
